# The interaction between long non-coding RNA LINC01564 and POU2F1 promotes the proliferation and metastasis of gastric cancer

**DOI:** 10.1186/s12967-022-03391-x

**Published:** 2022-05-13

**Authors:** Jixu Wang, Futao Hou, Lusheng Tang, Ke Xiao, Tengfei Yang, Zhiqiang Wang, Gu Liu

**Affiliations:** 1 Department of Interventional Vascular Surgery, Affiliated Hospital (Clinical College) of Xiangnan University and Key Laboratory of Medical Imaging and Artificial Intelligence of Hunan Province, Xiangnan University, 25 Renmin West Road, Chenzhou, 423000 Hunan China; 2 Department of General Surgery, Hunan Provincial People’s Hospital, First Affiliated Hospital of Hunan Normal University, 61 the West Jiefang Road, Furong District, Changsha, 410003 Hunan China; 3 Department of Gastroduodenal and Pancreatic Surgery, Hunan Cancer Hospital and The Affiliated Cancer Hospital of Xiangya School of Medicine, Central South University, Changsha, 410013 Hunan China; 4 Department of Gastrointestinal Surgery, Chenzhou First People’s Hospital and The First Affiliated Hospital of Xiangnan University, 102 Luojiajing, Chenzhou, 423000 Hunan China

**Keywords:** Gastric cancer, Metastasis, lncRNAs, LINC01564, POU2F1, Carcinogenic progression

## Abstract

**Background:**

An increasing number of studies have demonstrated that long non-coding RNAs (lncRNAs) serve as key regulators in tumor development and progression. However, only a few lncRNAs have been functionally characterized in gastric cancer (GC).

**Methods:**

Bioinformatics analysis was conducted to find lncRNAs that are associated with GC metastasis. RNA FISH, RIP, and RNA pull down assays were used to study the complementary binding of LINC01564 complementary to the 3′UTR of transcription factor POU2F1. The transcription activation of LINC01564 by POU2F1 as a transcription factor was examined by ChIP assay. In vitro assays such as MTT, cell invasion assay, and clonogenic assay were conducted to examined the impacts of LINC01564 and POU2F1 on GC cell proliferation and invasion. Experiments in vivo were performed to access the impacts of LINC01564 and POU2F1 on GC metastasis.

**Results:**

The results showed that LINC01564 complementary bound to the 3′UTR of POU2F1 to form an RNA duplex, whereby stabilizing POU2F1 mRNA and increasing the enrichment in cells. The level of LINC01564 was also increased by POU2F1 through transcription activation. In vitro assays showed that LINC01564 promoted the proliferation, invasion and migration of GC cells through increasing POU2F1. In vivo experiments indicate the promotion of GC proliferation and metastasis by the interaction between LINC01564 and POU2F1.

**Conclusion:**

Taken together, our results indicate that the interaction between LINC01564 and POU2F1 promotes the proliferation, migration and invasion of GC cells.

**Supplementary Information:**

The online version contains supplementary material available at 10.1186/s12967-022-03391-x.

## Introduction

It is estimated that approximately 90% of human genome DNA sequence is actively transcribed, but only 2% of the genes encode proteins. The majority of the transcripts that do not encode proteins are defined as non-coding RNAs (ncRNAs) [1]. Most of ncRNAs belong to long non-coding RNAs (lncRNAs) or microRNAs (miRNAs). lncRNAs are another type of regulatory ncRNAs. LncRNAs are usually longer than 200 nucleotides with lack of protein-coding capability, but lncRNAs can interact with proteins [2]. LncRNAs were previously considered as transcriptional noise, but recent studies have shown that lncRNAs play important roles in diverse epigenetic processes, including transcriptional regulation, histone modification, RNA splicing, and chromatin remodeling [3, 4]. LncRNAs have been shown to regulate a wide variety of cellular processes, including cell growth, stem cell differentiation, cell cycle, apoptosis, metabolism, and cancer progression [5–9].

Particularly, studies have demonstrated that lncRNAs are aberrantly expressed in a variety of cancers and play key vital roles in promoting tumor initiation and progression [10, 11]. Therefore, functional lncRNAs are potential targets for cancer diagnosis and prognosis. Gastric cancer (GC) is a considerable health issue worldwide. It is the fifth most frequently diagnosed cancer and one of the leading causes of death due to cancer in the world [12]. In China, GC is the second most frequently diagnosed cancer and the second leading causes of cancer death [13]. In 2018, over 1,000,000 cases of newly diagnosed cancer are GC. It is estimated that 783,000 people died of GC in 2018, equating to 1 in every 12 deaths globally. The outlook for patients with metastatic GC is unfavorable, with median survival usually less than 1 year [14, 15]. According to American Cancer Society, the 5-year relative survival rate is 69% for localized stage, 31% for regional state, and 5% for distant stage. Thereby, it is urgent to explore novel biomarkers and therapeutic targets for GC.

Several lncRNAs such as UCA1, MALAT1, HOXA11-AS, and ZEB1-AS1 have been proposed as potential diagnostic and prognostic biomarkers in GC [16]. Gao et al. have shown by meta-analysis that numerous lncRNAs are abnormally over-expressed in GC [17]. These lncRNAs includes AFAP1-AS1, ANRIL, CASC15, CCAT2, GAPLINC, H19, HOTAIR, HOTTIP, LINC00673, MALAT1, MEG3, PANDAR, PVT1, Sox2ot, UCA1, XIST, ZEB1-AS1 and ZFAS1, and the lncRNAs are associated with GC patient survival. Particularly, the expression levels of AFAP1-AS1, CCAT2, LINC00673, PANDAR, PVT1, Sox2ot, ZEB1-AS1 and ZFAS1 can be used to predict the GC prognosis. However, only a few lncRNAs that are associated with GC have so far been functionally characterized. Furthermore, the mechanism by which lncRNAs regulate GC remains to be fully elucidated.

LINC01564 is a lncRNA which is closely related to glutamate-cysteine ligase catalytic subunit [18]. LINC01564 has been shown to be associated with a variety of cancers. For example, Ke et al. found that high expression level of LINC01564 is associated with poor overall survival in testicular cancer patients [19]. Lee et al. reported that LINC01564 is elevated in prostate cancer patient samples and cell lines [20]. LINC01564 is also upregulated urine samples of prostate cancer patients compared to healthy individuals. The integrity of LINC01564 has been confirmed by 3′RACE and 5′RACE experiments [21].

In the present study, we aim to identify the function of lncRNA LINC01564 in promoting GC metastasis. In addition, we investigate whether LINC01564 affects the GC tumor progression through its interaction with transcription factor POU2F1. POU2F1, also known as OCT-1, has been reported to be associated with GC proliferation, migration, and epithelial-to-mesenchymal transition (EMT) via the enhancement of DLX6-AS1 expression by targeting the promoter region [22].

## Materials and methods

### Go annotation of survival-related genes

Gene Ontology (GO) enrichment analysis was performed on the up-regulated genes in metastatic GC. DAVID (The Database for Annotation, Visualization, and Integrated Discovery) software was used to annotate and visualize GO terms. The data used for Go enrichment analysis was from Gene Expression Profiling Interactive Analysis (GEPIA) and TCGA.

### Patients

Tumor samples and matched adjacent normal tissues were collected with informed consent from gastric cancer patients during surgeries performed between January 2019 to January 2020 at Hunan Cancer Hospital (Changsha, China). The current study was approved by the medical ethical review committees at Hunan Cancer Hospital, and written informed consent were obtained by all patients. There was no other therapy before surgery in recruited patients. The tumor tissues were immediately preserved in liquid nitrogen after surgery.

### Cell culture, RNA extraction, and real-time PCR

The human GC cell lines, including SGC-7901, MKN-45, BGC-823, and MKN-28, as well as the normal gastric epithelial cell line (GES-1) were purchased from American Type Culture Collection (ATCC). BGC823, MKN-45 and MKN28 cells were cultured in Roswell Park Memorial Institute (RPMI) 1640 medium (Invitrogen, Waltham, MA, USA); GES-1 and SGC7901 cells were cultured in a Dulbecco-modified Eagle medium (Invitrogen). All the media were supplemented with 10% fetal bovine serum (Sigma-Aldrich, St. Louis, MO, USA) and 100 U penicillin–streptomycin. Cells were cultured under 5% CO_2_ at 37 °C.

Total RNA was extracted from cells using Trizol reagent (TaKaRa Biotechnology, Dalian, China) according to the manufacturer’s protocol. Total RNA was reverse transcribed with a PrimerScript RT-PCR kit (Takara Biotechnology, Dalian, China). Real time qPCR was conducted using a standard SYBR Green PCR kit (Roche, Upper Bavaria, Germany) protocol with a CFX real-time instrument (Bio-rad, Hercules, CA, USA). The relative expression was calculated using the 2−∆∆Ct method. The transcription level of GAPDH was used as an internal control. The primer sequences are listed in Additional file 1.

### RNA FISH

The procedure was performed by using a Ribo FISH Hybridization Kit by RiboBio (Guangzhou, China). MKN-45 and SGC-7901 cells seeded on the glass coverslips (0.8 × 0.8 cm) were cultured to 60–70% confluence. The coverslips were washed with a solution of 0.5% Triton X-100 in 1× PBS. The coverslips were incubated with LINC01564, POU2F1 mRNA or U6 oligodeoxy-nucleotide probes by RiboBio (Guangzhou, China) with a hybridization solution containing 1% blocking solution in a humid chamber at 37 °C overnight. The following day, the coverslips were rinsed 3 times for 15 min (5 min each time) at 42 °C with a solution of 0.1% Tween-20 in 4× sodium citrate buffer (SSC), once for 5 min in 2× SSC and once for 5 min in 1× SSC in dark conditions. Finally, after rinsing with 1× PBS for 5 min 3 times at room temperature and re-staining by DAPI (Cell Signaling Technologies, Danvers, USA), the coverslips were observed and photographed using a fluorescent microscope (Leica, Wetzlar, Germany). Images were analyzed using Image-Pro Plus 6.0 software (Media Cybernetics, Rockville, USA). For double-fusion FISH, the protocol was similar. The only difference was that the coverslips were incubated with both LINC01564 and POU2F1 oligodeoxy-nucleotide probes.

### Immunofluorescence (IF)

Cells were seeded into 6-well plates with the autoclaved cover glasses placed at the bottom of the wells. After cells grown to 50% confluence on the cover-slips, PBS rinsing was performed, followed by 4% paraformaldehyde (PFA) fixation at RT for 10 min. After washing, cells were permeated with PBS which containing 0.5% Triton X-100 for 20 min and blocked with 3% bovine serum albumin (BSA, Thermo Fisher Scientific) for 1 h at RT. After rinsing with PBS, primary antibody, including anti-E-cad (#20874-1-AP, 1:1000; Ptgcn), and anti-Vimentin (#60330-1-Ig, 1:1000; Ptgcn) antibodies were added to the cells attached on the cover-slips at 4 °C for overnight incubation. Alexa Fluor® 488-conjugated secondary antibody (ab150077, Abcam) incubation was conducted at RT for 1 h. Then, the cell nuclei were counterstained with 4-6-diamidino-2-phenylindole (DAPI) reagent (Sangon Biotech) for 10 min and the slices were mounted. Finally, images were observed and photographed using a confocal microscope (Zeiss, Jena, Germany).

### Cell transfection

The short interfering RNA (siRNA) targeting LINC01564 were synthesized by RiboBio (Guangzhou, China). The siRNA sequences for LINC01564 were as follows: si-LINC01564-69: guide 5′-UUUCAGUUUGUUUUCCAUCCC-3′, passenger 5′-GAUGGAAAACAAACUGAAACU-3′; si-LINC01564-214: guide 5′-UGAUUUCUUGCCUUUUGAGCU-3′, passenger 5′-CUCAAAAGGCAAGAAAUCAGC-3′; si-LINC01564-506: guide 5′-UUUUGUCAAUAAAUAGGUGAU-3′, passenger 5′-CACCUAUUUAUUGACAAAAUU-3′. Oligonucleotide transfection was performed using Lipofectamine 2000 reagent (Invitrogen, United States). Short hairpin RNA (shRNA) directed against LINC01564 were synthesized by GeneChem (Shanghai, China) and were inserted into the vector. Lentiviral vector DNA and package vectors were transfected into HEK-293T cells. At 72 h after transfection, lentivirus supernatants were harvested and used to infect SGC-7901 and MKN-45 cells. Stable shRNA cell lines were generated following selection with 1 μg/mL puromycin.

### Protein extraction and Western blot analysis

Cells were washed three times with PBS and collected in RIPA lysis buffer (Beyotime Biotechnology, China) supplemented with a protease inhibitor cocktail (Calbiochem, United States). Protein concentration was determined by staining with Coomassie Blue (Beyotime Biotechnology, China). After electrophoresis, the protein was transferred to a polyvinylidene fluoride membrane (Merck Millipore, Germany). After blocking with 0.1% Tween 20 (TBS-T) in Tris-buffered saline containing 5% skim milk for 1 h at room temperature, the primary monoclonal antibody, including anti-POU2F1 (#10387-1-AP, 1:500; Ptgcn), anti-E-cad (#20874-1-AP, 1:1000; Ptgcn), anti-Vimentin (#60330-1-Ig, 1:1000; Ptgcn), and anti-β-actin (#66009-1-Ig, 1:2000; Ptgcn), was added to the membrane and incubated overnight at 4 °C. The next day, the membrane was incubated with secondary antibody Goat Anti Rabbit IgG/HRP for 1 h at room temperature and the signal was detected in a Bio-Rad ChemiDoc XRS imaging system. The ratio of the gray value of the target protein to the gray value of β-actin indicates the relative amount of protein.

### RNA stability assay

SGC-7901 and MKN-45 cells with stably expressed siRNAs against LINC01564, LINC01564 overexpression or NC were seeded into 6-well plates to get 50% confluency after 24 h. Cells were treated with 5 μg per mL actinomycin D and collected at indicated time points. The total RNA was extracted by miRNeasy Kit (Qiagen) and analyzed by RT-PCR. RNA stability profiling was generated from two biological replicates. The turnover rate and half-life of mRNA was estimated according to a previously published paper [23].

### RNA pull down

LINC01564 was in vitro transcribed and biotin‐labeled using the Biotin RNA Labeling Mix (Roche) and T7 RNA polymerase (Roche). After being treated with DNase I (Takara) to remove DNA and purified using RNeasy Mini Kit (Qiagen, Shenzhen, China), 3 µg of purified RNA was incubated with 1 mg of whole‐cell lysate from SGC-7901 and MKN-45 cells for 1 h at 25 °C. Next, the complexes were extracted by streptavidin agarose beads (Invitrogen) and the RNA present in the pull‐down material was detected by qPCR as described above.

### RNA immunoprecipitation (RIP)

SGC-7901 and MKN-45 cells were used to carry out RIP assay with the Magna RIP RNA‐Binding Protein Immunoprecipitation Kit (Millipore, Bedford, MA) and an AGO2 specific antibody (Millipore) following the instructions. RIP‐derived RNA was detected by qPCR as described above.

### Chromatin immunoprecipitation assay (ChIP)

ChIP assay was performed via a commercially purchased chromatin immunoprecipitation kit (Millipore, Temecula, CA), using anti-POU2F1 or anti-IgG antibodies. Cells were first cross-linked for 10 min by adding formaldehyde directly to culture medium to a final concentration of 1%. Cross-linked cells were then washed twice with cold PBS (with protease inhibitors), scraped, pelleted, resuspended in 200 μL SDS lysis buffer (1% SDS, 10 mM EDTA, 50 mM Tris–HCl, pH 8.0), and incubated for 10 min on ice. The lysates were then sonicated for five cycles of 30 s each, resting on ice for 1 min between cycles. After sonication, the samples were centrifuged and the supernatants diluted tenfold in ChIP dilution buffer with protease inhibitors. Cross-linked chromatin was incubated overnight with 5 μg anti-POU2F1 or anti-IgG of 1 mL at 4 °C. Antibody-protein-DNA complexes were isolated by immunoprecipitation. After extensive washing, pellets were eluted by freshly prepared elution buffer (1% SDS, 0.1 M NaHCO_3_). Formaldehyde cross-linking was reversed by 5–12-h incubation at 65 °C after adding 20 μL 5 M NaCl. Samples were purified through PCR purification kit columns (Qiagen, Chatsworth, CA) and used as a template in PCR. The primer sequences are listed in Additional file 1.

### DNA affinity precipitation assay (DAPA)

The 31-wt-S oligonucleotide biotinylated at the 5′ end (Operon Biotech., Inc.) was annealed with the anti-sense oligonucleotide. DAPA was performed as described previously [24] with some modifications. Briefly, the assays were done in a final volume of 400 μL of buffer D (20 mM HEPES, 10% glycerol, 50 mM KCl, 0.2 mM EDTA, 1.5 mM MgCl_2_, 10 μM ZnCl_2_, 1 mM DTT and 0.25% Triton X100, pH 7.9), by mixing 4 μg of biotinylated double stranded 31-wt oligonucleotides with 20–30 μg of nuclear extracts. The mix was incubated on ice for 45 min and then was added to the buffer D with equilibrated Streptavidin coated Magnetosphere particles (SMPs) (Promega). The mixture was incubated at room temperature for 2 h with continuous agitation. SMPs were then captured using the magnetic stand and the supernatant removed without disturbing the SMPs pellet. Particles were washed four times with the buffer D and the final pellet obtained was resuspended in 2× SDS-PAGE loading buffer and boiled for 5 min to uncouple the oligonucleoide bound proteins. After capturing the SMPs using the magnetic stand, the supernatant was loaded on SDS-PAGE gel and Western analysis was performed.

### MTT assay

Media was discarded from cell cultures. 50 µL of serum-free media and 50 µL of MTT solution were added into each well. The plate was incubated at 37 °C for 3 h. After incubation, 150 µL of MTT solvent was added into each well. The plate was wrapped in foil and shaken on an orbital shaker for 15 min. Absorbance was read at OD = 590 nm. The absorbance was proportional to cell number.

### Cell invasion assay

Cells were cultured to 70 to 80% confluency and sub-cultured into the invasion chamber. Cell invasion chambers were incubated overnight in a humidified tissue culture incubator at 37 °C, 5% CO_2_ atmosphere. Non-invading cells were removed. Cells in the invasion chambers were stained by adding 0.5 mL of each solution from the Diff-Quik kit. The permeable supports were subsequently transferred through each stain solution and the two plates of water. The permeable support membrane was allowed to air dry. The invaded cells were observed under the microscope.

### Colony formation assay

Exponentially growing cells were re-plate in dishes. The dishes were left in the incubator until cells in control dishes had formed sufficiently large clones. After treatment, the number of cells in the resulting cell suspension was counted using a Coulter counter, and the cell suspension was diluted in sterile tubes so that 100 or up to 104 cells after the treatment could be pipetted into the test wells. Cells were re-plated immediately after treatment. The dishes were then put in an incubator and leave there until cells in control dishes had formed sufficiently large clones. The medium above the cells was removed and the cells were rinse with PBS. After the PBS was removed, 2–3 mL of a mixture of 6.0% glutaraldehyde and 0.5% crystal violet were added. After 30 min, the glutaraldehyde crystal violet mixture was carefully removed, and the cells were rinsed with tap water. The dishes with colonies were allowed to dry in normal air at room temperature.

### Tumor xenograft in nude mice

SGC-7901 cells with POU2F1 (OE), POU2F1 (OE) plus LINC01564 (KD) or control vector, A total of 40 BALB/C nude mice of either gender (age: 4 weeks, weight: 18–22 g) (Hunan SJA Laboratory Animal Co., Ltd., Changsha, Hunan, China) were reared in the Specific Pathogen Free (SPF)-level condition and randomly classified into 3 groups with 4 mice in each group. The 3 groups were injected the cells as follows: (a) SGC-7901 cells with POU2F1 (OE); (b) SGC-7901 cells with POU2F1 (OE) and LINC01564 (KD); (c) SGC-7901 cells with control vector. All mice were euthanized by cervical dislocation at the end of experiment, and GC tumors were collected.

### Hematoxylin and eosin (HE) staining

Xenografted tumor tissues were fixed with 4% paraformaldehyde (BOSTER, Wuhan, China), embedded in paraffin, sectioned, and visualized at 40× and 100× magnification after HE staining. We analyzed the number of metastatic foci and the relative area of metastatic foci in the field of HE staining of each section, using the method described in the reference [25]. The area of metastatic lesions displayed by HE staining was measured by pathological graphic analysis software, and compared with the total HE staining area. The results were presented as a percentage (the total he area was set as 100%).

### Immunohistochemistry (IHC)

The IHC analyses was conducted as described previously [26]. Anti-MMP9 and anti-KI67 antibodies were used to detect the expression of MMP9 and KI67 in GC tissues.

### Statistical analysis

The statistical analysis for all data was analyzed using SPSS 21.0 software (IBM, Armonk, NY, USA). Each experiment was repeated 3 times. The measurement data were expressed as mean ± standard deviation. The comparison between two groups was analyzed by t test, and among multiple groups was analyzed by one-way analysis of variance (ANOVA) followed by post hoc Dunnett’s test. A P value < 0.05 was considered to be significantly different.

## Results

### Bioinformatics analysis implied a mutual interaction between POU2F1 and LINC01564

**Fig. 1:**
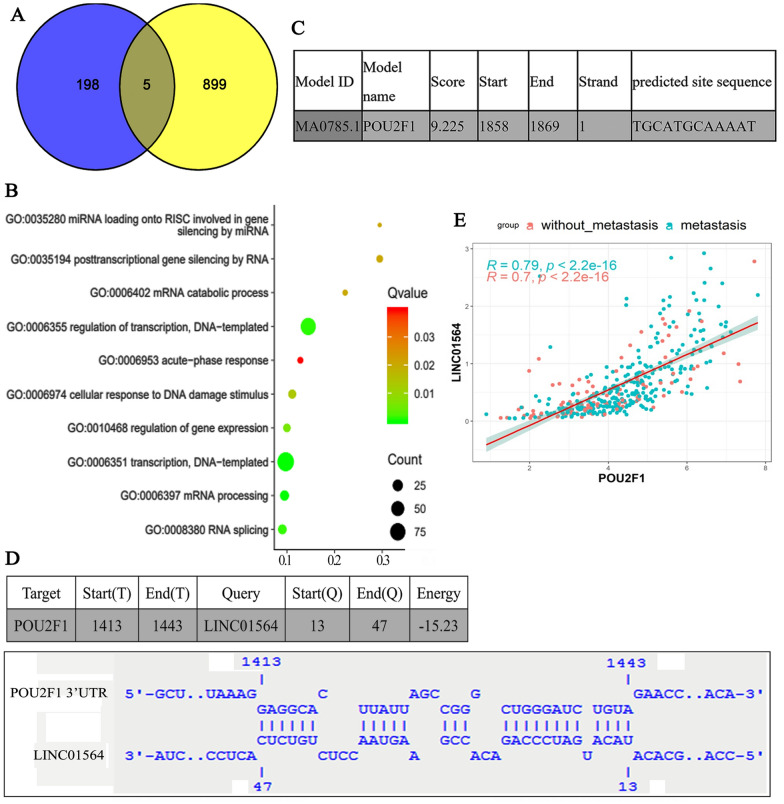
Bioinformatics analysis of lncRNAs that are potentially associated with GC metastasis. **A** The Venn diagram of overlapping differentially expressed lncRNAs among TCGA and GEPIA datasets. Venn diagrams shows differentially expressed genes shared by TCGA and GEPIA. A total of 5 lcnRNAs were identified in the intersection. **B** GO enrichment analysis of the up-regulated genes in metastatic GC. GO enrichment analysis was performed on the up-regulated genes in metastatic GC using DAVID. Several transcriptional regulation pathways such as GO: 0006351—transcription, DNA-templated and GO: 0006355—regulation of transcription, DNA-templated, were prominently involved. **C** Putative site of POU2F1 was predicted on LINC01564 promoter sequence. **D** Putative binding site of POU2F1 3′UTR and LINC01564. **E** Using data from TCGA database, we analyzed the correlation between POU2F1 and lncRNA LINC01564 in patients with or without GC metastasis

Human Cancer Metastasis Database (HCMDB) web (http://hcmdb.i-sanger.com/statistics) provides a platform to analyze the data in the Cancer Genome Atlas Stomach Adenocarcinoma (TCGA) database. The HCMD-EXP00440 platform contains the information of 372 GC samples, among which 21 cases were metastatic while 351 cases were non-metastatic. A total of 224 ncRNAs were differentially expressed in metastatic GC compared to those in non-metastatic GC (*P* < 0.05, and log2 [fold changes] > 1 or log2 [fold changes] < − 1). Among the 224 ncRNAs, 203 were up-regulated in metastatic GC, while 21 were down-regulated. GEPIA web (http://gepia.cancer-pku.cn/) serves to analyze genes whose expression is associated to the prognosis of GC. We conducted an intersection analysis using the data from HCMD-EXP00440 and GEPIA webs (Fig. 1A). In detail, the up-regulated genes in metastatic GC were overlapped with the genes that were associated to the prognosis of GC. Five lncRNAs (CATIP-AS2, TTC3-AS1, LINC01993, LINC01564 and LINC02015) were identified in the overlapping region.

Based on the data from the HCMD-EXP00440, we found that the mRNA level of 603 genes were significantly up-regulated in metastatic GC compared to those in non-metastatic GC (*P* < 0.05). The 603 genes were used for Gene Ontology (GO) enrichment analysis using DAVID (The Database for Annotation, Visualization, and Integrated Discovery) software. Results indicated these genes primarily impact biological processes responsible for gene transcription, such as GO: 0006351—transcription, DNA-templated, and GO: 0006355—regulation of transcription, DNA-templated (Fig. 1B). Among the 603 genes, there are eleven transcription factors, including POU5F1, CUX2, TBX19, POU2F1, PLAG1, ZFP57, LMX1A, MZF1, BACH1, ONECUT1, and IRF9, which are implicated in above two biological processes.

We hypothesized that a few of these transcription factors might be involved in the modulation of the expression above-mentioned five lncRNA. A transcription prediction web, JASPAR (http://jaspar.genereg.net/collection/core/) implied that LINC01564 is a potential target of POU2F1 (Fig. 1C). Furthermore, bioinformatics analysis (http://rna.informatik.uni-freiburg.de/IntaRNA/Input.jsp) indicates that the 3′UTR of mRNA is complementary to LINC01564, thus LINC01564 might form an RNA duplex with POU2F1 mRNA (Fig. 1D). This RNA duplex might impact the stability of POU2F1 mRNA, and consequently regulate the POU2F1 protein level. Using data from TCGA database, we analyzed the correlation between POU2F1 and lncRNA LINC01564 in patients with or without GC metastasis. We found that POU2F1 expression is positively correlated to LINC01564 in all patients with (*p* < 0.001, r = 0.79) or without GC metastasis (*p* < 0.001, r = 0.7, Fig. 1E). Therefore, we conjectured a positive feedback regulation, in which POU2F1 probably promotes LINC01564 expression by a transcriptional activation; LINC01564, in turn, increases POU2F1 by stabilizing the mRNA. Our following study aimed to identify their mutual interaction and further study their roles in GC.

### Elevated expression of LINC01564 and POU2F1 predicts poor clinical outcomes in GC patients

**Fig. 2:**
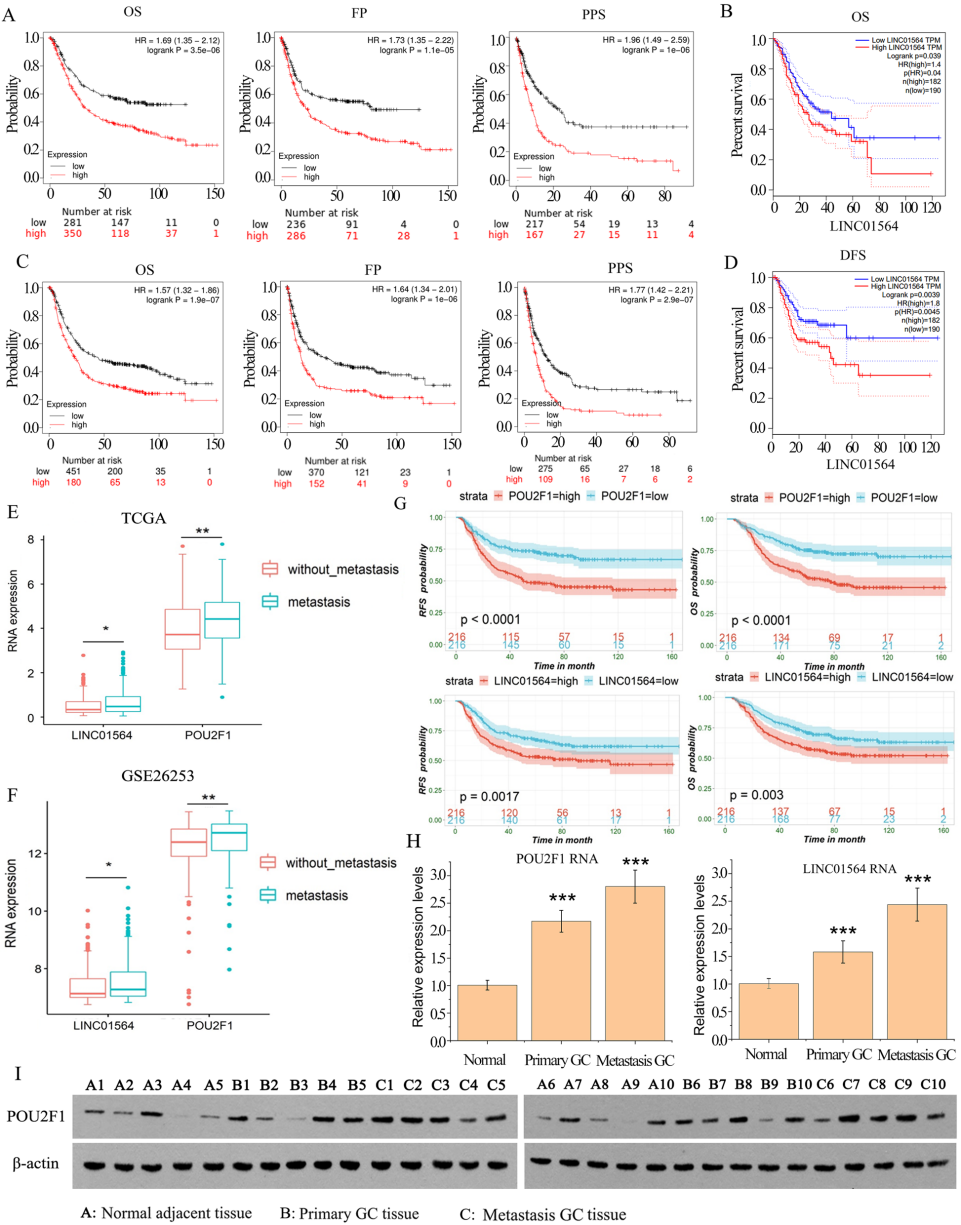
Elevated expression of LINC01564 predicts poor clinical outcomes in GC patients. The mRNA expression of LINC01564 was stratified into high or low expression. The association of LINC01564 expression with the progression of GC was analyzed using the Kaplan–Meier survival curves (**A**) and GEPIA (**B**); similarly, the association of POU2F1 expression with the progression of GC was analyzed using the Kaplan–Meier survival curves (**C**) and GEPIA (**D**). Using data from TCGA database, we analyzed the expression of POU2F1 and lncRNA LINC01564 in patients with or without GC metastasis (**E**). Moreover, we analyzed the expression of POU2F1 and LINC01564 using the data from GSE26253 dataset (**F**). As indicated by the results from GSE26253 dataset, high expression of LINC01564 and POU2F1 are associated to poor OS and recurrence free survival (RFS) (**G**). The RNA levels of LINC01564 and POU2F1 in normal adjacent tissues (n = 10), primary GC tissues (n = 10), and metastasis GC tissues (n = 10) were analyzed using qPCR (**H**) and Western blot (**I**). Significant differences are indicated by *p < 0.05, **p < 0.01 and ***p < 0.001

We analyzed the prognostic effects of LINC01564 and POU2F1 expressions on GC patients with Kaplan–Meier Plotter database at https://kmplot.com/analysis/ and GEPIA web. We selected the median value to divide the samples into high and low expression groups. The result showed that higher LINC01564 expression was significantly associated with shorter overall survival (OS), first progression (FP), post progression survival (PPS), and disease-free survival (DFS) in GC patients (Fig. 2A, B). Higher mRNA expression of POU2F1 was significantly associated with shorter OS, FP and PPS in GC patients, as well (Fig. 2C, D).

Using data from TCGA database, we analyzed the expression of POU2F1 and LINC01564 in patients with or without GC metastasis. We found that LINC01564 and POU2F1 expression are significantly higher in patients with GC metastasis than in patients without GC metastasis (Fig. 2E). Moreover, we analyzed the expression of POU2F1 and LINC01564 using the data from GSE26253 dataset. In consistent to the data from TCGA database, LINC01564 and POU2F1 expression are significantly higher in patients with GC metastasis than in patients without GC metastasis (Fig. 2F). As indicated by the results from GSE26253 dataset, high expression of LINC01564 and POU2F1 are associated to poor OS and recurrence free survival (RFS) (Fig. 2G). We collected GC tissues and sorted the GC tissues to the metastatic and non-metastatic. The expression levels of LINC01564 and POU2F1 in normal adjacent tissues (n = 10), primary GC tissues (n = 10), and metastasis GC tissues (n = 10) were tested using qPCR and Western blotting. The RNA levels of both LINC01564 and POU2F1 were highest in metastasis GC tissue and lowest in normal adjacent tissue (Fig. 2H). Similarly, the protein level of POU2F1 was highest in metastasis GC tissue and lowest in normal adjacent tissue (Fig. 2I). The results indicated that higher levels of LINC01564 and POU2F1 may correlate with GC metastasis.

### The expression pattern and distribution of LINC01564 in GC cell lines

**Fig. 3:**
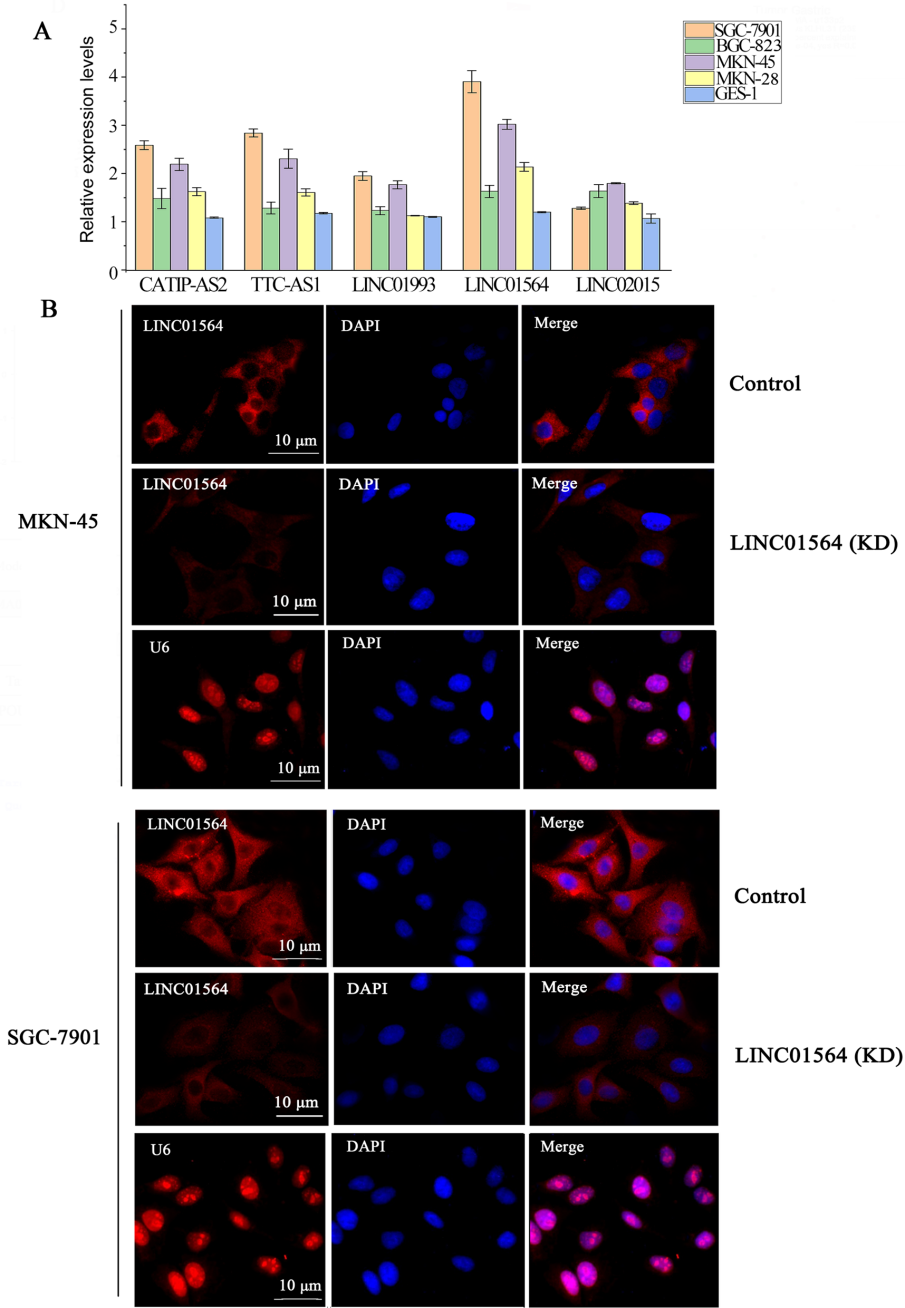
The expression pattern and distribution of LINC01564 in GC cell lines. **A** The expression levels of lncRNAs CATIP-AS2, TTC3-AS1, LINC01993, LINC01564, and LINC02015 in SGC-7901, BGC-823, MKN-45, MKN28, and GES-1 were analyzed by qPCR. **B** The subcellular distribution of LINC01564 and the IF intensity were detected by RNA Fluorescent in situ hybridization (FISH) in MKN-45 cells and SGC-7901 with LINC01564 knockdown or not. U6 was the positive control for the nucleus

According to HCMDB, five lncRNAs (CATIP-AS2, TTC3-AS1, LINC01993, LINC01564 and LINC02015) are highly expressed in GC tissue. The expression levels of these lncRNAs in different GC cell lines were further analyzed by PCR assay. LINC01564 was highly expressed in all of the GC cell lines (Fig. 3A). SGC-7901 and MKN-45 cells expressed the highest and the second highest levels of LINC01564 among the tested cell lines. Next, the subcellular distribution of LINC01564 in MKN-45 and SGC-7901 cells were visualized by RNA Fluorescent in situ hybridization (FISH). In both cell lines, LINC01564 was distributed primarily in cytoplasm (Fig. 3B), suggesting that LINC01564 might exert a post-transcriptional regulation function. After LINC01564 knockdown, the fluorescent of LINC01564 probe was notably decreased.

### LINC01564 regulated GC cell proliferation invasion and EMT

**Fig. 4:**
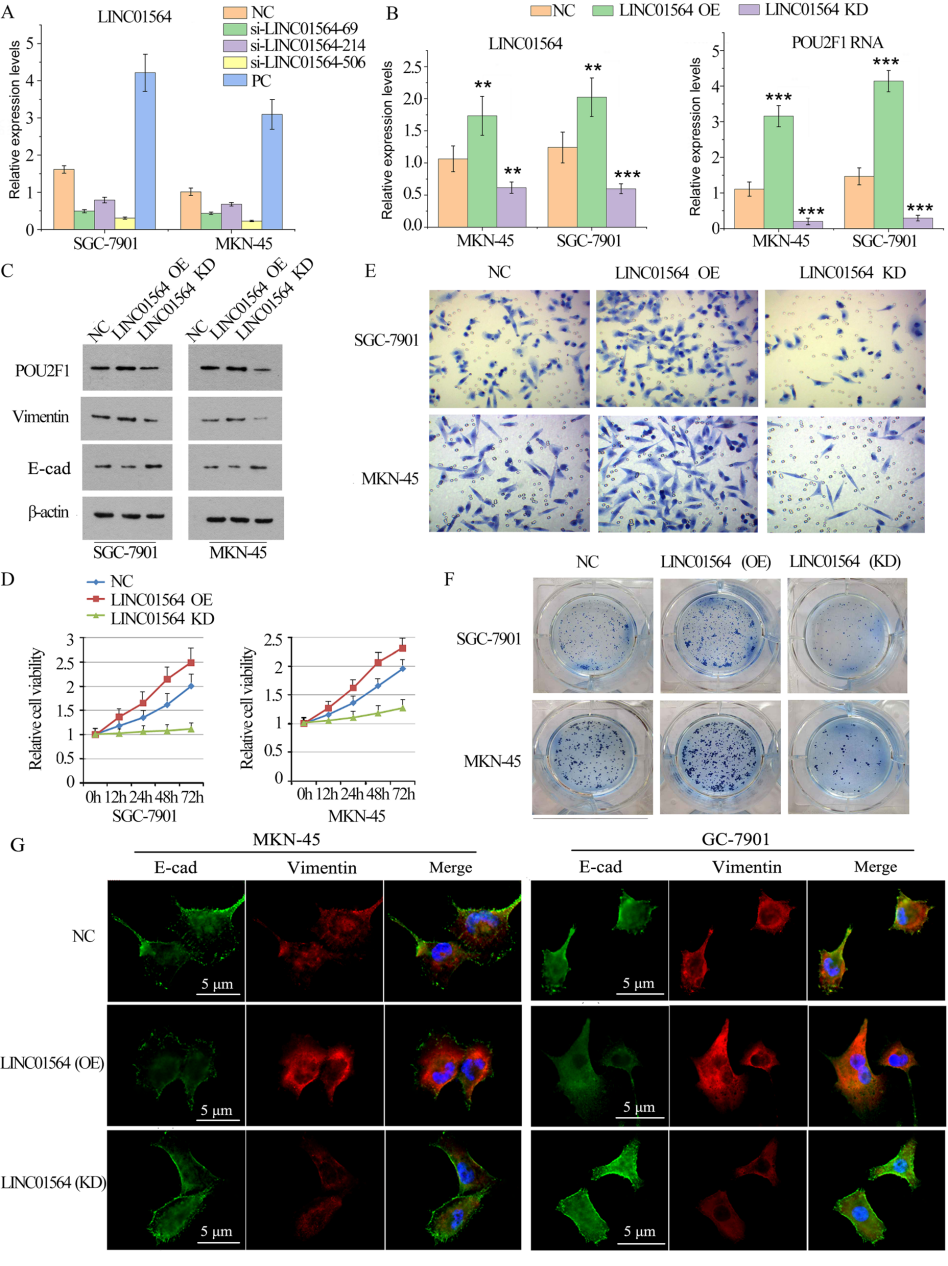
LINC01564 regulated GC cell proliferation invasion and EMT. **A** The RNA level of LINC01564 was detected by qPCR. SGC-7901 and MKN-45 cells were transfected with the negative control (NC), si-LINC01564-69, si-LINC01564-214, si-LINC01564-506, as well as LINC01564 overexpression vector (Positive control, PC). **B** The RNA levels of LINC01564 (left) and POU2F1 (right) were detected by qPCR after LINC01564 overexpression and knockdown. **C** The protein levels of POU2F1, E-cadherin, Vimentin, and β-actin were analyzed using Western blot. **D** MTT assay was conducted in SGC-7901 (left) and MKN-45 (right) cells. X axis represents time, while Y axis represents cell proliferation rate. Cell proliferation rate was measured at 0 h, 12 h, 24 h, 48 h, and 72 h time points. **E** Cell invasion assay was conducted in SGC-7901 and MKN-45 cells transfected with NC, LINC01564 (OE), or LINC01564 (KD). **F** Colony formation assay was conducted in SGC-7901 and MKN-45 cells transfected with NC, LINC01564 (OE), or LINC01564 (KD). **G** The expression levels of E-cadherin and Vimentin were further analyzed by IF. Significant differences are indicated by **p < 0.01 and ***p < 0.001

To determine the roles of LINC01564 in GC metastasis, we designed 3 siRNAs that targets the positions of 67–89, 214–236, and 506–528 of LINC01564, respectively. The 3 siRNAs were defined as si-LINC01564-69, si-LINC01564-214, and si-LINC01564-506. As shown in Fig. 4A, all of the siRNAs worked well in either cell line, with si-LINC01564-506 having the highest silencing efficiency. Si-LINC01564-506 was picked to knockdown (KD) LINC01564 in further experiments. In addition, SGC-7901 and MKN-45 cells transfected with LINC01564 overexpression (OE) plasmid to promote LINC01564 expression. Cells transfected with empty vector were served as the negative control. When LINC01564 was overexpressed, the RNA and protein levels of POU2F1 also increased. However, knockdown of LINC01564 is associated with the reduction of POU2F1 RNA and protein levels (Fig. 4B). Furthermore, the protein levels of EMT-induced markers, including E-cadherin, and Vimentin were analyzed (Fig. 4C). LINC01564 overexpression down-regulated E-cadherin and up-regulated Vimentin. While LINC01564 KD up-regulated E-cadherin and down-regulated Vimentin. The cell viability, invasion and colony formation ability were enhanced with the overexpression of LINC01564 (Fig. 4D–F). However, these properties of GC cells were suppressed with LINC01564 knockdown. The expression levels of E-cadherin and Vimentin were further analyzed by IF (Fig. 4G). Compared with NC group, LINC01564 OE significantly increased the expression of Vimentin, but decreased the expression of E-cadherin. LINC01564 KD decreased the expression of Vimentin, while increased the expression of E-cadherin. IF results were in accordance with those of Western blotting. Our findings suggested that LINC01564 promotes GC cell proliferation and invasion in vitro.

### LINC01564 stabilizes POU2F1-RNA in GC cells by forming a duplex

**Fig. 5:**
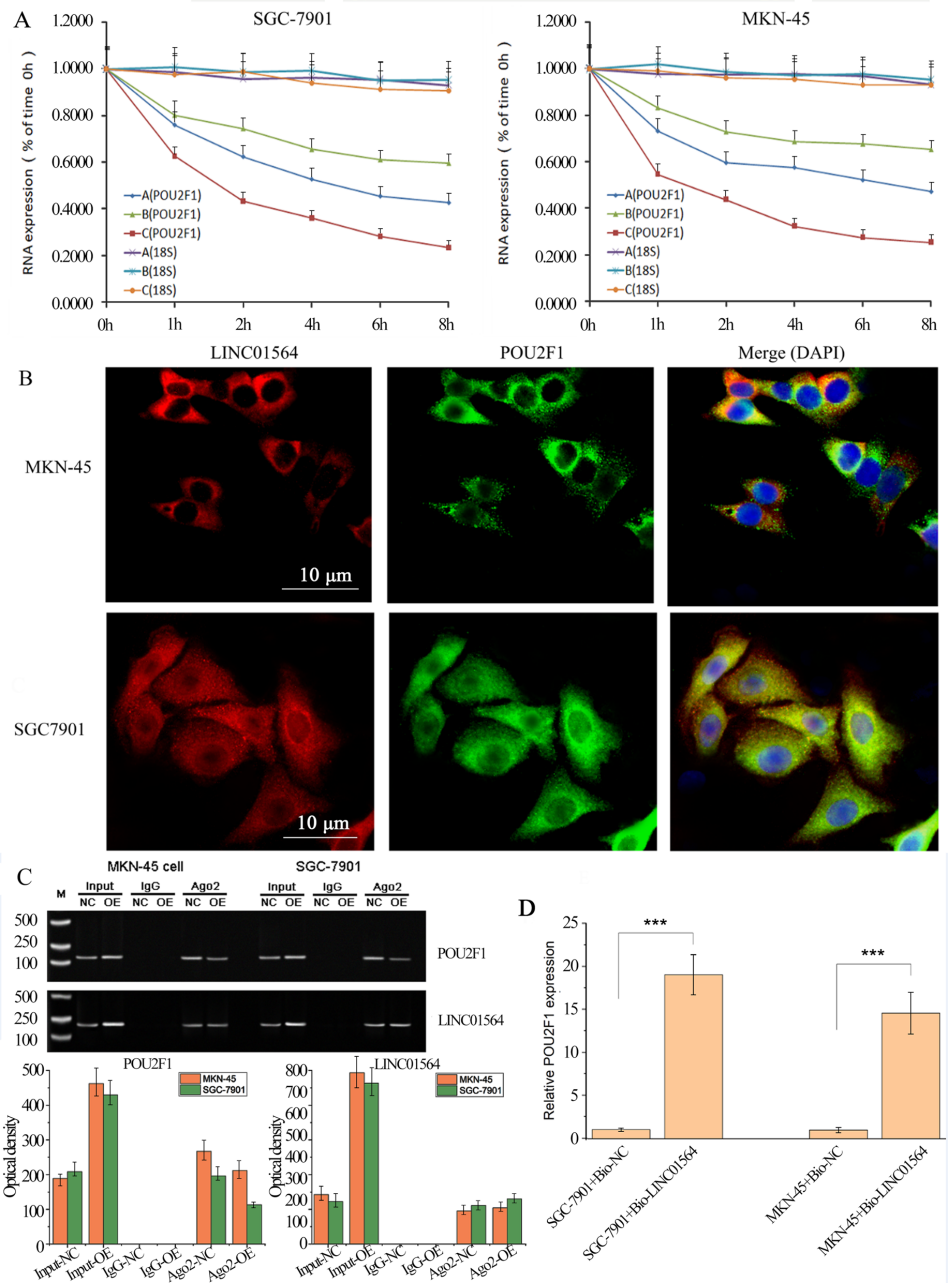
LINC01564 directly interacts with POU2F1 RNA. **A** RNA stability assay of POU2F1 and 18S in SGC-7901 (left) and MKN-45 (right) cells. **A** Represents NC. **B** Represents LINC01564 OE. **C** Represents LINC01564 KD. Cells were treated with 5 μg per ml actinomycin D and collected at 0, 1, 2, 4, 6, and 8 h. The subcellular localizations of LINC01564 and POU2F1 were visualized by RNA FISH in MKN-45 cells (**B**) and SGC-7901 (**C**). DAPI was used to stain nuclei. **D** Western Blot analysis of proteins RIP with anti-Ago2 or anti-IgG antibody in SGC-7901 and MKN-45 cells. **E** RNA pull-down assay using bio-NC or Bio-LINC01564 probe in SGC-7901 and MKN-45 cells. Significant differences are indicated by ***p < 0.001

Bioinformatics analysis (http://rna.informatik.uni-freiburg.de/IntaRNA/Input.jsp) unveiled an interaction between POU2F1 3′UTR (from 1413 to 1443) and LINC01564 (from 47 to 13) through complementary base pairing. This interaction probably prevents a few of miRNAs targeting POU2F1 3′UTR, whereby increasing the stability of POU2F1 mRNA (Fig. 1D). To demonstrate the interaction between LINC01564 and POU2F1, we firstly conducted RNA stability assay in SGC-7901 and MKN-45 cells. Cells were transfected with LINC01564 OE, LINC01564 KD or NC. Transfected cells were then treated with actinomycin D and collected at 0, 1, 2, 4, 6, and 8 h. The RNA stability of POU2F1 was analyzed, and 18S RNA was used as an intrinsic control. The RNA stabilities of 18S in different groups were similar (Fig. 5B). While POU2F1 RNA was more stable in LINC01564 OE cells and less stable in LINC01564 KD cells, indicating that LINC01564 significantly stabilized POU2F1 RNA. We further conducted RNA FISH assay in the two cell lines and found that LINC01564 and POU2F1 co-localized in cell plasma (Fig. 5C).

RNA immune-precipitation (RIP) assay was conducted to confirm the interaction between LINC01564 and POU2F1. SGC-7901 and MKN-45 cells were transfected with LINC01564 OE or NC. Then the cells were lysed and RNA was extracted. RIP assay was conducted on the RNA extraction. It is found that in both cell lines, compared to NC cells, the signal of POU2F1 in Ago2 fraction was weaker in LINC01564 OE cells (Fig. 5D). It indicated that there was less POU2F1 RNA in Ago2 fraction in LINC01564 OE cells. Combined with the RNA stability assay and FISH assay, the results showed that LINC01564 may form a duplex with POU2F1 and thus preventing POU2F1-RNA from being degraded by RISC. Ago2 protein is the main component of the RNA silencing complex, RISC. RNA degradation is mainly processed by miRNA-induced RISC. Therefore, our results suggested that the RNA duplex formed by POU2F1-RNA and LINC01564 reduced the degradation of POU2F1-RNA by RISC.

To confirm the direct binding of LINC01564 on POU2F1 RNA, RNA pull-down assay was conducted next. In either cell line, POU2F1 RNA level of LINC01564 pull-down group was significantly higher that of NC, indicating that LINC01564 directly interact with POU2F1 RNA (Fig. 5E).

### POU2F1 activates LINC01564 transcription and promotes GC proliferation and invasion

**Fig. 6:**
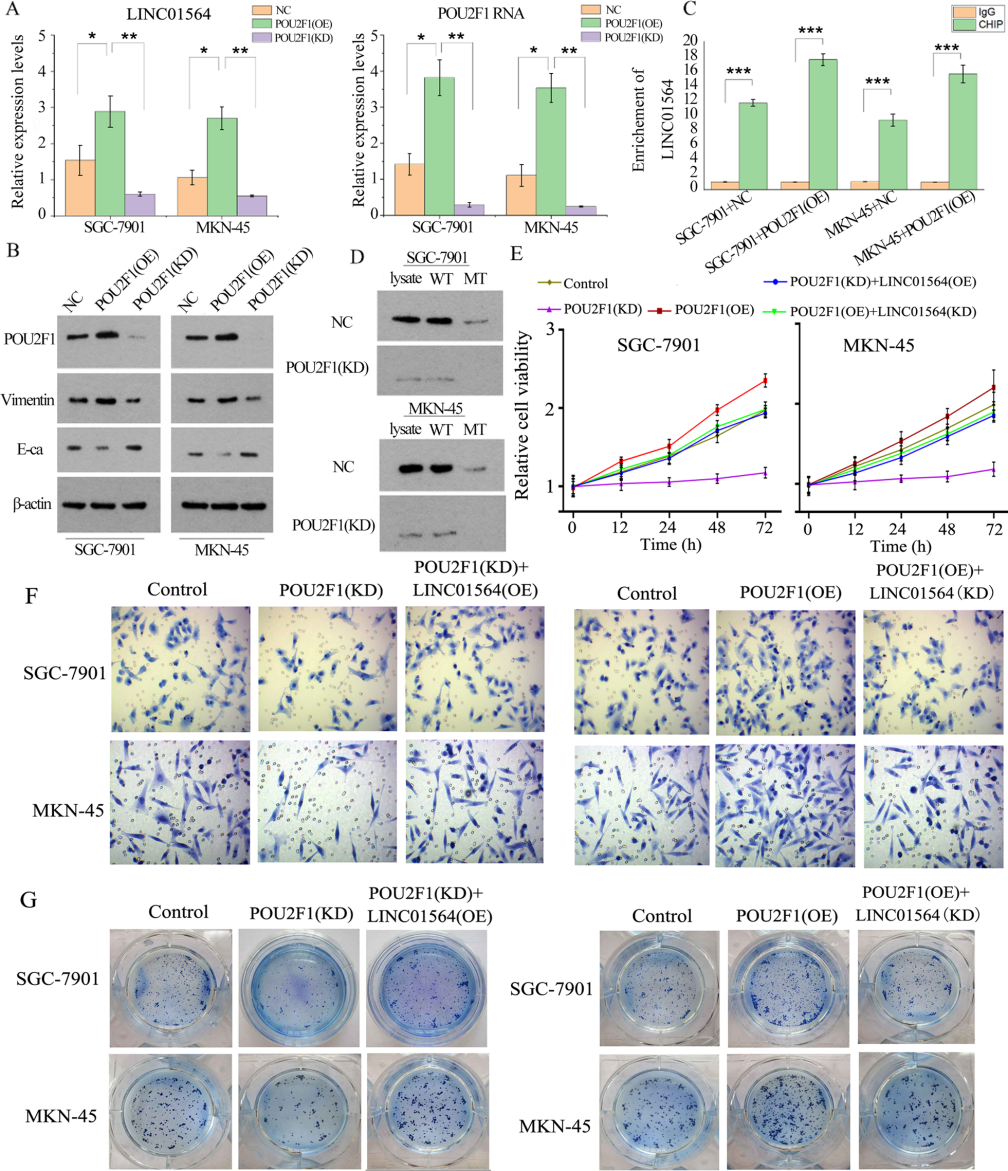
POU2F1 activates LINC01564 transcription and promotes GC proliferation and invasion. **A** The RNA levels of POU2F1 (left) and LINC01564 (right) were detected by qPCR after POU2F1 overexpression and knockdown. **B** The protein levels of POU2F1, E-cadherin, Vimentin, and β-actin were analyzed using Western blot. Group a, b, and c represent NC, POU2F1 OE, and POU2F1 KD, respectively. **C** ChIP assay was conducted in SGC-7901 and MKN-45 transfected with POU2F1 OE or NC. Cells were divided into IgG and ChIP groups. In ChIP group, POU2F1 was pulled down and the promoter sequence of LINC01564 was detected using qPCR. IgG and Poly-II were used as the negative and positive controls, respectively. **D** Biotinylated DNA pull-down assay was conducted in SGC-7901 and MKN-45 cells using WT or MT LINC01564 promoter probe. SDS-PAGE gel and Western analysis of POU2F1 were performed. SGC-7901 and MKN-45 cells transfected with NC, POU2F1 (OE), POU2F1 (KD), POU2F1 (OE) + LINC01564 (KD) or POU2F1 (KD) + LINC01564 (OE). Then cells were subjected to MTT (**E**), invasion (**F**) and colony formation assays (**H**). Significant differences are indicated by *p < 0.05, **p < 0.01 and ***p < 0.001

We further investigated whether POU2F1 affected LINC01564 expression in turn. SGC-7901 and MKN-45 cells were transfected to overexpress or knock down POU2F1, and the RNA levels of POU2F1 and LINC01564 were analyzed using qPCR. As shown in Fig. 6A, B, in POU2F1 OE cell, the RNA levels of both LINC01564 and POU2F1 were higher than NC. In POU2F1 KD cells, the RNA levels of both LINC01564 and POU2F1 were lower than NC. Since a putative binding site of POU2F1 on LINC01564 promoter sequence was predicted, ChIP assay was conducted to further confirm the prediction. SGC-7901 and MKN-45 cells were transfected with POU2F1 OE or NC. In ChIP assay, anti-POU2F1 antibody was used to extract POU2F1 protein together with DNA binding to the protein. As indicated by qPCR, the promoter sequence of LINC01564 was detected in the binding DNA (Fig. 6C). Overexpression of POU2F1 increased the enrichment of LINC01564 promoter sequence in the protein-DNA complex. In addition, DNA affinity precipitation assay (DAPA) was conducted to check affinity of DNA (the LINC01564 promoter sequence) with POU2F1 protein. The results showed that POU2F1 was successfully pulled down by WT LINC01564 promoter probe but not by the MT probe (Fig. 6D).

Results from MTT, cell invasion, colony formation and Western blot assays showed that POU2F1 overexpression promoted cell viability, invasion, colony formation ability and EMT of GC, while POU2F1 knockdown conversely suppressed these GC properties (Fig. 6E–G). When POU2F1 was knocked down, GC cell viability, invasion, colony formation ability and EMT (Fig. 6E–G). LINC01564 knockdown abolished the cancer promoting effects conferred by overexpressed POU2F1.

### The cancer-promoting effects of POU2F1 are dependent on LINC01564

**Fig. 7:**
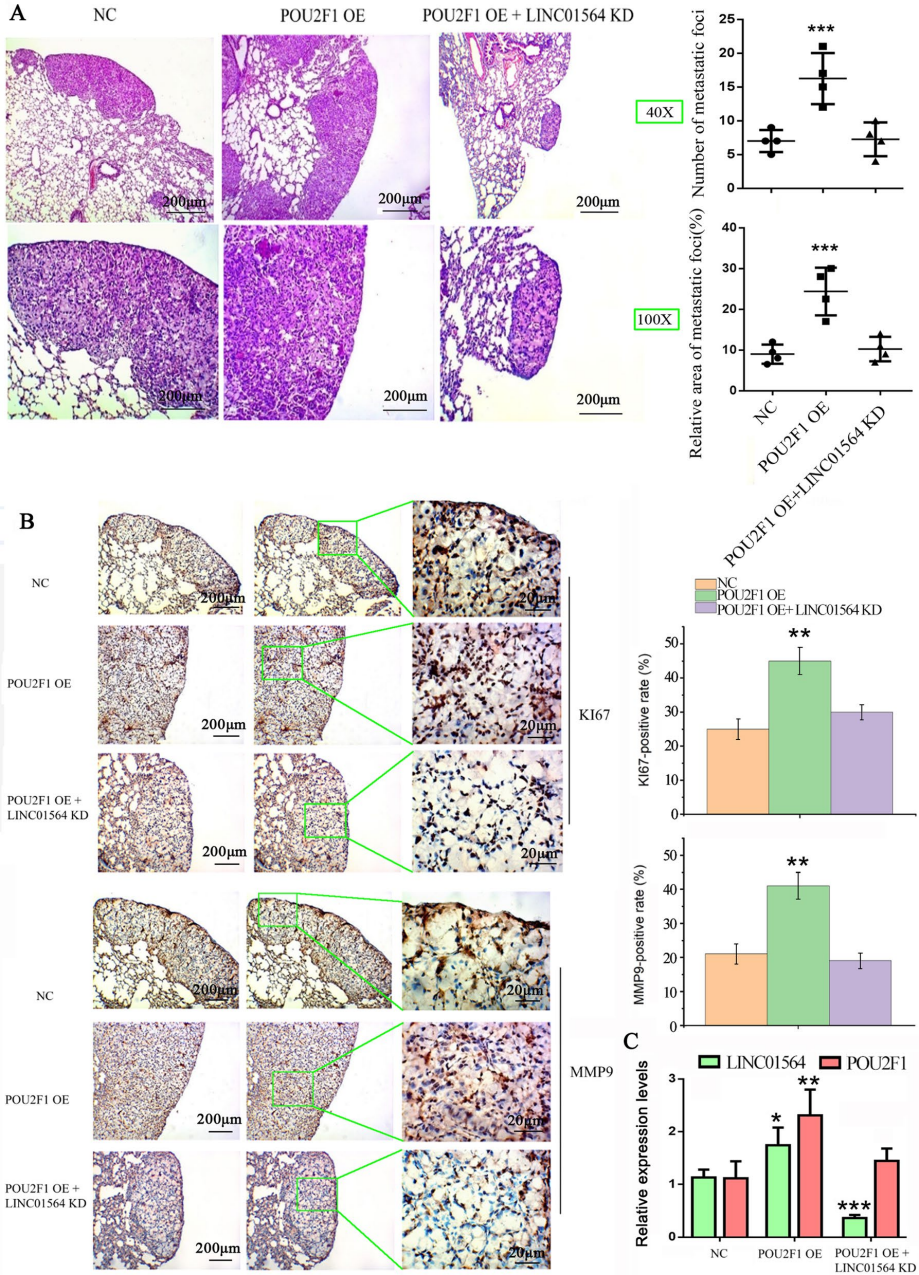
The impact of POU2F1 and LINC01564 on GC metastasis in vivo. Analysis of an experimental metastatic animal model was performed by injecting SGC-7901 cells stably transfected with POU2F1 (OE), POU2F1 (OE) plus LINC01564 (KD), or empty vector into nude mice (n = 4). **A** Tumor tissues from the mice in each group were stained with H&E staining. The number and relative area of metastatic foci was shown and quantified. **B** Tumor tissues from the mice in each group were stained with anti-KI67 and anti-MMP9 antibodies in immunohistochemistry assay. **C** PCR assay was performed to detect the expression levels of LINC01564 and POU2F1 in the collected tumor tissues. Significant differences are indicated by *p < 0.05, **p < 0.01 and ***p < 0.001

Our study in vitro confirmed that the cancer-promoting effects of POU2F1 is dependant on LINC01564. To study the effects of POU2F1 and LINC01564 on the metastatic ability of GC cells in vivo, we firstly transfected SGC-7901 cells with POU2F1 (OE), POU2F1 (OE) plus LINC01564 (KD) or control vector, and then injected the transfected cells into mice through the tail vein. In order to clearly present pathological features of tumor, we took pictures at the same location in the sections of HE and IHC staining. This is the method advocated by the pathological examination. Through HE staining, you can see the tissue structure, the location, size and shape of tumor in lung tissues; Through IHC staining, you can observe the expression levels of target proteins at the same place shown in the pictures of the HE staining. By observing same location in the pictures of HE and IHC staining, we can fully analyzed the pathological features of tumor. HE staining showed that the tumors formed in the POU2F1 (OE) group were substantially larger than those in the control group (Fig. 7A), while LINC01564 knockdown suppressed the growth of tumor with POU2F1 (OE). Moreover, we analyzed the number of metastatic foci and the relative area of metastatic foci in the field of HE staining of each section. POU2F1 (OE) mice had more metastatic foci and bigger area of metastatic foci than control mice. The tumor-promoting properties of POU2F1 OE was partially rescued by LINC01564 knockdown. GC formed from stably POU2F1 (OE)-transfected SGC-7901 cells exhibited increased positivity for KI67 and MMP9 than GC from the control cells (Fig. 7B). Compared with the POU2F1 (OE) group, when LINC01564 was co-transfected, the tumors were smaller and GC cells exhibited decreased positivity for KI67 and MMP9. As indicated by PCR assay, POU2F1 overexpression was associated with increased LINC01564 in GC tumor (Fig. 7C). LINC01564 knockdown not only decreased LINC01564 expression, but also inhibited POU2F1 expression in GC tumor induced by POU2F1 overexpression vector. These findings indicate that POU2F1 promotes the metastatic ability of GC cells in vivo, and the knockdown of LINC01564 inhibits POU2F1 expression and metastasis.

## Discussion

So far, the clinical treatment of GC remains limited. It is urgent to explore novel biomarkers and therapeutic targets for GC. Several lncRNAs, such as UCA1, MALAT1, HOXA11-AS, and ZEB1-AS1 have been reported to play important roles in GC [16]. However, only several lncRNAs have been functionally characterized in GC metastasis. The molecular mechanisms exerted by lncRNAs are diverse. In this study, we systematically integrated GC clinical information and gene expression data from TCGA and GEPIA. By comparing cancer samples with normal samples, we identified 5 GC-related lncRNAs, including CATIP-AS2, TTC3-AS1, LINC01993, LINC01564 and LINC02015. Predicted using JASPAR database, we identified that the level of LINC01564 is associated with GC metastasis and LINC01564 thus may serve as a potential biomarker for diagnosis and prognosis of GC. By exploring the associations between expression of LINC01564 and clinicopathological features in GC patients, we found that the expression of LINC01564 was associated with GC metastasis. This study for the first time confirmed the interaction between LINC01564 and POU2F1.

Using INTARNA algorithm as described by Mann et al. [27], which enables fast and accurate prediction of RNA–RNA hybrids by incorporating seed constraints and interaction site accessibility, we predicted the binding site of LINC01564 on POU2F1-mRNA. Using immunoprecipitation and RNA pull down assay, we confirmed that LINC01564 complementary bond to the 3′UTR of transcription factor POU2F1 to form an RNA duplex and thus stabilizing POU2F1 and increasing POU2F1 protein level. Due to positive feedback, the level LINC01564 also increased. We hypothesize that the binding of LINC01564 to the 3′UTR of POU2F1 prevents the mRNA of POU2F1 from binding miRNAs and thus stabilizing the mRNA. Collectively, our findings suggested that LINC01564 interacted with POU2F1 mRNA 3′UTR and increased POU2F1 level by stabilizing its mRNA. Similar mechanism has also been reported in previous studies. Jia et al. have demonstrated that long noncoding RNA PXN‐AS1‐L interacts with SAPCD2 3′UTR and stabilizes SAPCD2 mRNA [28]. PXN‐AS1‐L thus promotes the malignancy of nasopharyngeal carcinoma cells via upregulation of SAPCD2. Yue et al. found that Down syndrome cell adhesion molecule antisense RNA 1 (DSCAM-AS1) binds to the 3′UTR mRNA of dCTP pyrophosphatase 1 (DCTPP1) and thus inhibits the binding of miRNAs [29]. Moreover, we found that POU2F1, in turn, modulates the transcription of LINC01564. The results indicate the tumorigenic activity of POU2F1 in GC and the association with the increase of LINC01564. Similarly, Xie et al. have found that the expression of POU2F1 is regulated by long non-coding RNA TUG1 and their interaction is associated with tumorigenesis of human osteosarcoma [30].

In vitro and in vivo experiments were performed to access the impacts of LINC01564 and POU2F1 on GC proliferation and metastasis. The results showed that LINC01564 promoted the proliferation, invasion and migration of GC cells. Previous studies highlighted the positive role of EMT in tumor metastasis, meanwhile lncRNAs could modulate cancer metastasis via affecting EMT [22]. Particularly, we further measured the expression level of EMT markers. The results demonstrated that LINC01564 could regulate GC cells metastasis via affecting EMT. In vivo assays showed that tumor with higher expression of POU2F1 had stronger staining of ki67 and produced more MMP9. ki67 is an important mark to determine cell proliferation especially in tumor tissue. MMP9 is a member of the MMP family. Studies have shown that MMP9 is overexpressed in various cancers and is related to tumor metastasis and invasion [31–35]. However, silencing of LINC01564 significantly reversed the oncogenic roles of POU2F1 in GC in vitro and in vivo, which supported that the cancer-promoting effects of POU2F1 was associated with LINC01564. Collectively, the results supported that POU2F1 was an important mediator of the roles of LINC01564 in GC. Functional experiments further revealed that overexpression of LINC01564 promoted GC cell proliferation, migration, and invasion in vitro*.* While LINC01564 knocking down repressed GC cell proliferation, migration, and invasion.

Similarly, several recent studies also show that the interactions between long non-coding RNAs and protein-coding genes are associated with cancer cell proliferation and metastasis. For example, the investigation by Jia et al. has shown that long non-coding RNA PXN-AS1-L promotes the malignancy of nasopharyngeal carcinoma cells via upregulation of SAPCD2 [28].

This study has some limitations. Although it has been demonstrated that LINC01564 promotes GC proliferation and metastasis via POU2F1, the involvement of other miRNAs or transcription factors cannot be excluded. Further research is needed to address the question.

In summary, our results indicate that the interaction between LINC01564 and POU2F1 promotes the proliferation, migration and invasion of GC cells through positive feedback. By systematically integrating bioinformatics and experimental methods, our findings firstly revealed that LINC01564 and POU2F1 have important oncogenic functions in GC. Our study provides novel insights on the functional characterization of LINC01564 in GC. In addition, LINC01564 is probably a potential biomarker for diagnosis and prognosis of GC in future studies.

## Supplementary Information

**Additional file 1: Table S1.** Primer for PCR assay. Primer for ChIP assay.

## Data Availability

The datasets generated/analyzed in the present study are available upon reasonable request from the corresponding author.
