# Supplementary material for: The interaction between long non-coding RNA LINC01564 and POU2F1 promotes the proliferation and metastasis of gastric cancer
Source: J Transl Med. 2022 May 13;20:220. doi: 10.1186/s12967-022-03391-x (PMC9101833; doi:10.1186/s12967-022-03391-x)
Supplement: Supplementary file 1 — Additional file 1: Table S1. Primer for PCR assay. Primer for ChIP assay. [file 12967_2022_3391_MOESM1_ESM.docx]

| Homo /Gene name | Sequence (5′--3′) |
| --- | --- |
| POU2F1-F | ATGAACAATCCGTCAGAAACCAG |
| POU2F1 -R | GATGGAGATGTCCAAGGAAAGC |
| CATIP-AS2-F | CTGTTCACCTGTGCTTTCCC |
| CATIP-AS2 -R | TATGGCCCTGTGTCCTTCTG |
| TTC3-AS1-F | AAGGGCATCAAAAGCTTCGG |
| TTC3-AS1-R | GTCCTACACCAGCATCCGTA |
| LINC01993-F | TCATCTGGCTTATGGCACAG |
| LINC01993-R | AGTAAGGGCTCTGTGTGCC |
| LINC01564-F | CCCAGACACCGAAGTAACCT |
| LINC01564-R | GTGGTGGACAGGGCTGAATA |
| LINC02015-F | GTGGTCAGGTGTGTGTGTTC |
| LINC02015-R | ATCAACATGCCATCCACAGC |
| β-actin-F | ACCCTGAAGTACCCCATCGAG |
| β-actin-R | AGCACAGCCTGGATAGCAAC |
| Primers U1 -F | GGGAGATACCATGATCACGAAGGT |
| Primers U1 -R | CCACAAATTATGCAGTCGAGTTTCCC |
| Primers 18S -F | CAGCCACCCGAGATTGAGCA |
| Primers 18S -R | TAGTAGCGACGGGCGGTGTG |

Primer for PCR assay

| Homo /Gene name | Sequence (5′--3′) |
| --- | --- |
| LINC01564-F | GCTGTCACCCTGTTGAAACC |
| LINC01564-R | CCCCAGGTGCTATAACTGCT |
| GAPDH-F | TACTAGCGGTTTTACGGGCG |
| GAPDH-R | TCGAACAGGAGGAGCAGAGAGCGA |

1. Primer for ChIP assay
